# Configurable, wearable sensing and vibrotactile feedback system for real-time postural balance and gait training: proof-of-concept

**DOI:** 10.1186/s12984-017-0313-3

**Published:** 2017-10-11

**Authors:** Junkai Xu, Tian Bao, Ung Hee Lee, Catherine Kinnaird, Wendy Carender, Yangjian Huang, Kathleen H. Sienko, Peter B. Shull

**Affiliations:** 1 State Key Laboratory of Mechanical System and Vibration, School of Mechanical Engineering, Shanghai Jiao Tong University, Shanghai, 200240 People’s Republic of China; 2 Department of Mechanical Engineering, University of Michigan, Ann Arbor, MI USA; 3 Department of Biomedical Engineering, University of Michigan, Ann Arbor, MI USA; 4 Vestibular Testing Center, Department of Otolaryngology, University of Michigan, Ann Arbor, MI USA

**Keywords:** Wearable systems, Gait retraining, Balance training

## Abstract

**Background:**

Postural balance and gait training is important for treating persons with functional impairments, however current systems are generally not portable and are unable to train different types of movements.

**Methods:**

This paper describes a proof-of-concept design of a configurable, wearable sensing and feedback system for real-time postural balance and gait training targeted for home-based treatments and other portable usage. Sensing and vibrotactile feedback are performed via eight distributed, wireless nodes or “Dots” (size: 22.5 × 20.5 × 15.0 mm, weight: 12.0 g) that can each be configured for sensing and/or feedback according to movement training requirements. In the first experiment, four healthy older adults were trained to reduce medial-lateral (M/L) trunk tilt while performing balance exercises. When trunk tilt deviated too far from vertical (estimated via a sensing Dot on the lower spine), vibrotactile feedback (via feedback Dots placed on the left and right sides of the lower torso) cued participants to move away from the vibration and back toward the vertical no feedback zone to correct their posture. A second experiment was conducted with the same wearable system to train six healthy older adults to alter their foot progression angle in real-time by internally or externally rotating their feet while walking. Foot progression angle was estimated via a sensing Dot adhered to the dorsal side of the foot, and vibrotactile feedback was provided via feedback Dots placed on the medial and lateral sides of the mid-shank cued participants to internally or externally rotate their foot away from vibration.

**Results:**

In the first experiment, the wearable system enabled participants to significantly reduce trunk tilt and increase the amount of time inside the no feedback zone. In the second experiment, all participants were able to adopt new gait patterns of internal and external foot rotation within two minutes of real-time training with the wearable system.

**Conclusion:**

These results suggest that the configurable, wearable sensing and feedback system is portable and effective for different types of real-time human movement training and thus may be suitable for home-based or clinic-based rehabilitation applications.

## Background

Postural balance and gait training are important for treating functional impairments. For example, balance rehabilitation can decrease dizziness in the elderly and patients with vestibular loss [1, 2], and gait training can enable individuals with knee osteoarthritis to reduce knee pain and knee loading [3] as altered gait patterns such as changing the foot progression angle (FPA) can reduce knee loads [4, 5] and can improve mobility post-stroke [6].

Wearable sensing systems have become an increasingly attractive option for standing balance and gait-related applications, because they are portable and relatively inexpensive as compared with non-portable, laboratory-based systems. However, current systems are in general designed for monitoring but not training movement as they do not have the capability of providing real-time feedback [7, 8]. Yigit et al. [9] presented a wearable soft sensing suit to estimate hip, knee, and ankle sagittal plane joint angles to non-invasively monitor the motion of impaired individuals in unrestricted settings. Donath et al. [10] presented a body-worn inertial sensor system to estimate stride length, stride time and cadence in real-time to allow clinicians and other health professionals to assess gait patterns related to functional limitations due to neurological or orthopedic conditions. Rodríguez-Martín et al. [11] introduced an inertial wearable system to analyze trunk movements for long-term monitoring of Parkinson’s symptoms outside of clinical settings. Guo et al. [12] presented an inertial system to estimate knee joint angles, identify gait cycles and evaluate balance and knee extensibility for individuals with hemiplegic gait. Wearable sensing systems typically provide kinematic information for diagnosing and monitoring, though movement training still primarily relies on therapist/physician observation and judgment [13].

Wearable feedback systems can enable automated and precise motor control for postural balance and gait training [14, 15] in persons with intact cognition and sensorimotor systems. Among the possible feedback modalities, visual and audio feedback can be effective for training human movement but can also potentially inhibit or overload the auditory and visual sensory channels [16]. Human skin is a good information receptor and thus haptics can also be effective for training and rehabilitation [14]. Vibrotactile feedback systems have been used as physical non-interrupting interfaces for movement training [17–19] since they are generally considered to be effective, small and economical. Vibrotactile feedback systems have applications in posture and gait training for individuals with age-related balance declines [20], individuals with vestibular [21] and neurological disorders [22] and knee osteoarthritis [23] and various rehabilitation applications as it is effective, small and economical [16]. A TactaPack wearable vibrotactors system has shown potential to reduce injuries during therapy due to improper patient joint movements [24]. Reeder et al. [25] presented a vibrotactile system that provides feedback to reduce knee hyperextension during gait in patients suffering from cerebral vascular accidents. A waist-worn vibrotactile system (Vertiguard-RT, Vesticure GmbH, Germany) with four stimulators on the front, back, left and right side of hip to improve balance training in patients with Parkinson’s disease [22] and olders [26].

Existing wearable inertial sensing and haptic feedback systems are typically designed to measure and train a single kinematic parameter for a single application. For example, a cell phone based sensory feedback system has been designed for balance rehabilitation training, where trunk tilt was measured via a single smartphone accelerometer and tactors plugged into the smartphone audio jack provided vibrotactile feedback cues [27]. Similarly, a wearable real-time posture corrective system was designed to integrate vibrotactile feedback with a wobble board system to improve posture control by enhancing ankle proprioception [28]. In addition, many rehabilitation applications involve correcting or restoring gait patterns [3]. Thus, a configurable sensing and feedback system could enable a wider array of training paradigms for postural balance and gait tasks.

The purpose of this research was to present a proof-of-concept reconfigurable wearable sensing and feedback system design for human movement training; this system is best suited for individuals with intact cognitive, sensory and motor systems, e.g., general balance disorders and knee osteoarthritis. The system is based on distributed nodes that can each be configured for sensing and/or feedback for various movement training applications. System hardware design and software control architecture are first introduced, and then postural balance and gait training experiments and experimental results are presented to demonstrate usability and feasibility of real-time feedback movement training for disparate applications.

## Methods

The highest priority requirements for the system were the abilities to provide configurable sensing and feedback across various locations on the body for posture and gait movements and to allow unencumbered motion. The hardware and software design described below were based on these requirements.

### Hardware design

**Fig. 1:**
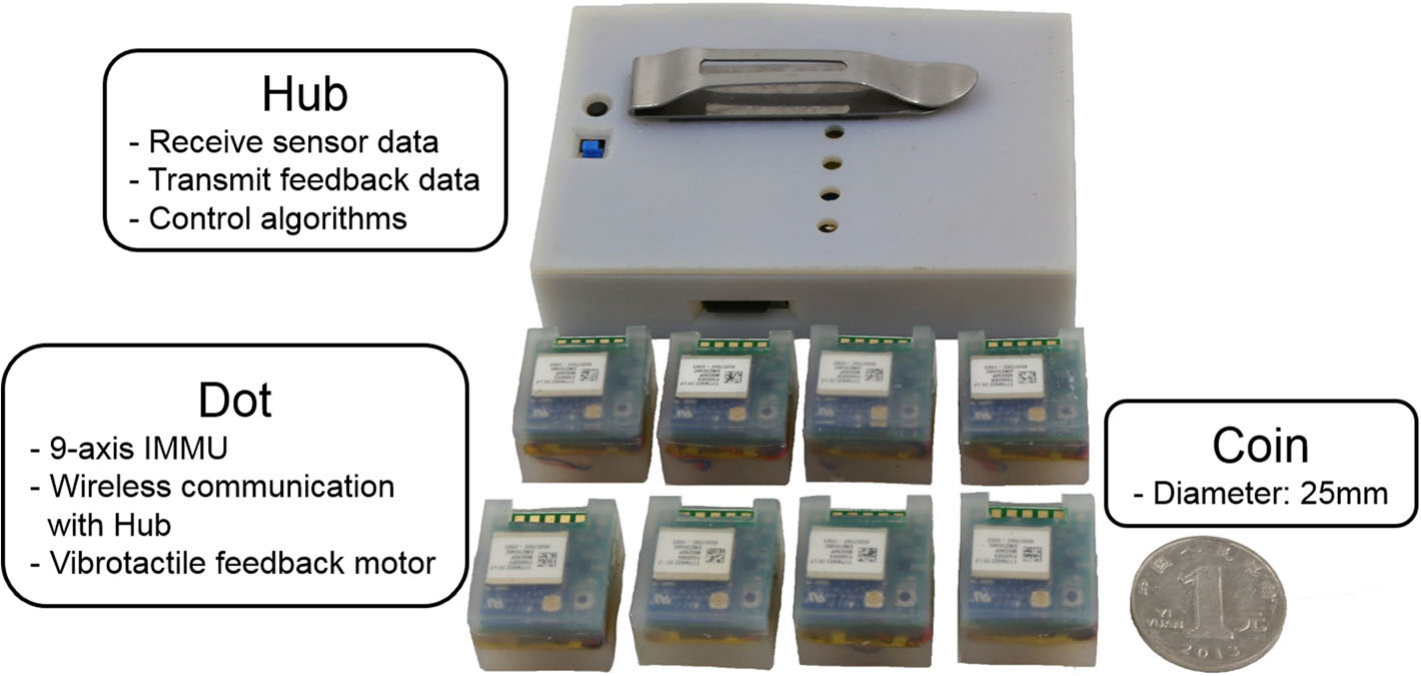
Wearable system hardware. Eight distributed nodes (Dots) simultaneously send and receive data to and from the central control unit (Hub) where real-time computation and control algorithms are performed. Dots are configured in custom software to act as sensing Dot, feedback Dot, or both. Each Dot is comprised of a 9-axis inertial measurement magnetometer unit (IMMU) sensor, vibrotactile feedback motor, ZigBee wireless communication module, and a 100 mAh lithium ion battery. All Dot components are embedded in a single silicon mold. Each Dot weighs about 12 g and the overall top surface area is roughly the same as a 25 mm coin

The wearable sensing and feedback system consists of eight distributed nodes (Dots) and a central control unit (Hub) that wirelessly connects to the Dots (Fig. 1). Each Dot can be configured for sensing and/or feedback according to the requirements of each specific application. The Hub receives sensor data from the Dots, performs control and algorithm computation, and then transmits feedback commands back to the Dots.

Sensing on each Dot is performed via a 9-axis inertial measurement magnetometer unit (IMMU) (MPU-9150, InvenSense, USA) and feedback via a flat eccentric vibrotactor motor (XY-B1034-DX, Xiongying Electronics, China) (Fig. 1). The vibrotactor vibrates at approximately 220 Hz, which is near the peak sensitivity frequency region for human skin [29], and the vibration strength of the vibrotactor is 1 g. Sensor data are wirelessly transmitted via a ZigBee module (EMZ3048C, MXCHIP, China) at 50 Hz and data is processed on the Microcontroller Unit (STM32W108, ST, Italy). Each Dot is powered by a 100 mAh lithium-ion battery (1.5 h of continuous use), and all of the Dot’s components are packaged together with silicone into a single module (Fig. 1). The overall size and weight of each Dot is 22.5 × 20.5 × 15.0 mm and 12 g, respectively.

The Hub was designed to receive sensor data from all Dots, process the control algorithm, and then transmit the feedback commands back to the Dots. The Hub includes three ZigBee modules which are connected to the MCU (STM32F401RB, ST, Italy) to enable higher speed data transfer when connecting simultaneously to multiple Dots. Data is stored on a 2 GB Micro-SD card (capable of storing 100 h of data). The overall size and weight of the Hub is 95 × 65 × 20 mm and 95 g, respectively, and it was designed to clamp to a waistband or be placed in a pocket. The total raw material cost of this system was approximately 250 US dollars.

### Software architecture

**Fig. 2:**
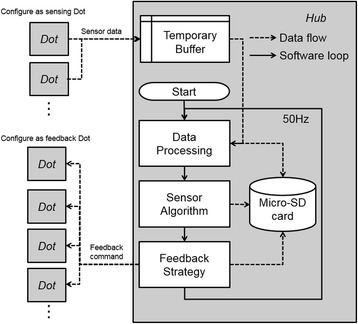
Dots can be configured in software as a sensing Dot, feedback Dot or both sensing and feedback Dot. The Hub receives sensor data and calculates the movement parameters of interest via a sensor fusion algorithm and then sends feedback commands to the feedback Dots according to the feedback strategy

Software structure and system data flow are shown in Fig. 2. In the Start block, the hardware and wireless connections are initialized and algorithm parameters (e.g. filter cutoff frequencies and algorithm feedback gains) are set according to the configuration file stored on the Micro-SD card. After the Start block finishes, the Hub begins receiving raw data from the Dots, which are then stored in a temporary buffer. In the Data processing block, data filtering and sensor calibration transformations are performed after reading in raw data from the buffer. Magnetometer data is calibrated via the ellipsoid fit method [30]. Accelerometer data and gyroscope data are filtered via a configurable low-pass filter. In the Sensor Algorithm block, human movement parameters (e.g., trunk sway or foot progression angle) are estimated with the processed sensor data via sensor fusion algorithms [31]. In the Feedback Strategy block, feedback commands are computed according to the movement parameters and feedback strategy. The sensor algorithm and the feedback strategy are configurable depending on the required application. Finally, the feedback commands are sent to the Dots. All data are stored on the Micro-SD card, including: raw sensor data from the Temporary Buffer, processed data from the Data Processing block, the movement parameters from the Sensor Algorithm block and feedback commands from the Feedback Strategy block. Sensor data, feedback commands, and the software loop were all updated at 50 Hz.

### Postural balance task experimental testing

Two usability and feasibility studies were performed to evaluate the proof-of-concept wearable system. In the first study, four healthy older adults (69.5 ± 3.5 years, two females/two males) were recruited to participate. The University of Michigan Institutional Review Board approved the experimental protocol (HUM00020302), which conformed to the Declaration of Helsinki. Informed consent was obtained from each participant prior to the start of the experiment.

**Fig. 3:**
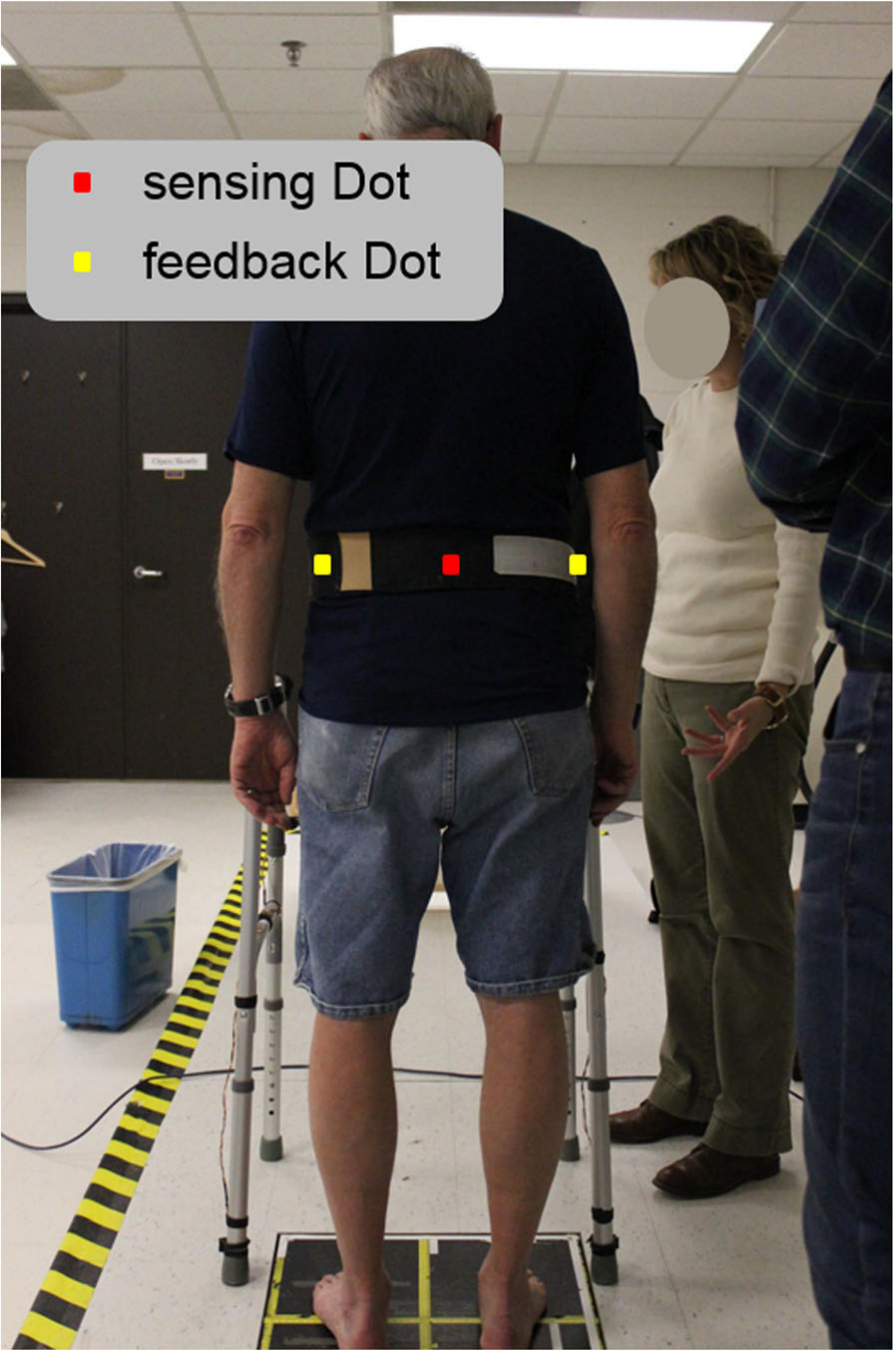
The sensing Dot was placed on the spine at approximately the L4/L5 level near the body center of mass and feedback Dots on the left and right sides of the torso at approximately the L4/L5 level. Dots were mounted inside a belt and secured around each participant’s waist

**Fig. 4:**
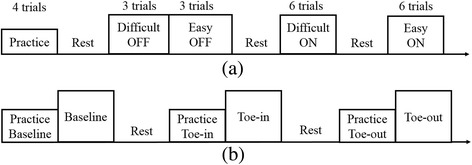
Experimental protocols of (**a**) posture balance task and (**b**) gait retraining task. The small box represents a practice trial and big box represents a training trial

Participants were initially asked to perform 2–5 exercises commonly used for balance rehabilitation [32] on firm and foam surfaces. Two participant-specific training exercises were selected to provide the participants with an appropriate level of balance challenge (not too easy and not too difficult) and were based on three factors: 1) if participants could perform the exercise for at least 30 s, 2) if participants rated the exercise as at least a 2 (I feel a little unsteady) out of 5 (I lost my balance) based on a visual analog scale [33], and 3) if participants’ medial-lateral (M/L) trunk tilt was greater than 1 degree for at least 80% of the 30-s trial. Those two exercises were categorized as easy or difficult based on the percentage of time trunk tilt was less than 1 degree. The trunk tilt is defined as the orientation of the trunk relative to the vertical gravitational vector, and medial-lateral trunk tilt was trained in this experiment. Feedback Dots were placed on the left and right sides of the torso at approximately the L4/L5 level, and a sensing Dot was placed at the spine at approximately the L4/L5 level (Fig. 3). Participants were acclimated to the system by practicing the two selected exercises twice (30-s trials). Continuous vibrotactile feedback with the same vibrational intensity and frequency was provided when trunk tilt exceeded the vibrotactor activation threshold (no feedback zone) and no feedback was provided when trunk tilt was inside the no feedback zone. They were instructed to move away from the vibration (i.e., align their body with the vertical gravity vector) to correct their posture in response to the vibrotactile feedback. The feedback threshold (Proportional-Derivative control of M/L trunk tilt angle plus half of the trunk tilt angular velocity (P + 0.5D)) was set to 1 degree as in previous studies [34]. Four 30-s trials were performed before the training section to give participants opportunity to familiar with the device and understand the instructions. The training protocol consisted of a battery of eighteen 30-s trials: three trials for each exercise without feedback (Off), followed by six trials for each exercise with feedback (On) (Fig. 4a). After each group of six trials, participants sat down and rested for 2 min or longer if requested.

To evaluate the performance of the balance trials, the root mean square (RMS) of trunk tilt and percentage time inside the no feedback zone were calculated. Two-way repeated measures ANOVA were performed for both RMS and percentage time inside the no feedback zone with feedback (Off vs. On) and difficulty of exercise (easy vs. difficult) as independent variables. For each condition, a value averaged for trials was used for analysis. Statistical significance was defined as *p* < 0.05, and data analysis was performed using MATLAB “(The MathWorks, Natick, MA) and SPSS (IBM, Ammonst, USA).”

### Gait retraining task experimental testing

**Fig. 5:**
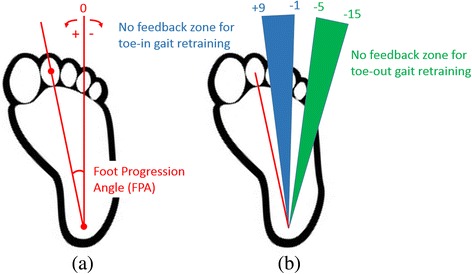
**a** The foot progression angle (FPA) is defined as the angle between the line from the calcaneous to the second metatarsal and the line of progression averaged from heel strike to toe off during the stance phase of walking for each step (toe-in angle is positive and toe-out angle is negative). **b** Participants were trained to walk with a new gait by adjusting their FPA to fall within the no feedback zone. Medial vibrotactile stimulus was given when FPA was greater than the inward threshold, lateral vibrotactile stimulus was given when FPA was less than the outward threshold, and no stimulus was given when FPA was between the two thresholds

In the second experiment, six healthy community dwelling older adults (72.5 ± 6.0 years, three females/three males) performed a gait retraining task. Participants with a body mass index (BMI) less than 30 were recruited to participate in the study if they were able to walk unassisted for longer than 30 min and could rotate their foot internally or externally while walking. The purpose of this task was to demonstrate if the wearable system could be used to train participants to alter their FPA during gait, which is an important kinematic measurement related to knee loading and pain for persons with knee osteoarthritis [3, 4]. The FPA is defined as the angle between the line from the calcaneous to the second metatarsal and the line of progression averaged from heel strike to toe off during the stance phase of walking for each step (Fig. 5a); a previously validated algorithm was used to estimate the FPA [35]. For this experiment, one sensing Dot was adhered to the dorsal side of the foot and one feedback Dot was placed on the medial and lateral sides of the mid-shank.

Initially a baseline walking trial on the treadmill with no feedback was performed to determine each participant’s baseline FPA. The feedback system was then used to train participants to adopt two separate gait patterns: a toe-in gait (foot internally rotated) and a toe-out gait (foot externally rotated), each training trial was followed a practice trial (Fig. 4b). The FPA was estimated during stance phase (heel-strike to toe off) and vibrotactile feedback was given to cue participants to adjust their FPA on the subsequent step. Continuous vibrotactile feedback with the same vibrational intensity and frequency was provided from the toe-off event through the end of the heel stride when FPA exceeded the no feedback zone threshold. This cue informed participants to adjust their FPA on the subsequent step and no feedback was provided when their FPA remained inside the no feedback zone. For training toe-in gait, a vibrotactile stimulation on the lateral side of shank occurred when the FPA was less than −1 degree and a vibrotactile stimulation on the medial side occurred when the FPA was larger than 9 degrees (Fig. 5b). For toe-out gait, the feedback thresholds were −15 degrees and −5 degrees (Fig. 5b). No feedback was given when the FPA did not exceed either threshold (no feedback zone). Each training trial lasted two minutes. All trials were performed on a treadmill and the walking speed was 1.0 m/s. A one-way ANOVA was performed to determine if there were any differences in FPA and the percentage of time inside the no feedback zone among baseline, toe-in, and toe-out gait conditions; in the case of a difference, Tukey’s post hoc analysis was used to determine whether there were differences between various pairs of gait patterns. Statistical significance was defined as the *p* < 0.05, and all data analysis was performed using MATLAB (The MathWorks, Natick, MA).

## Results

### Posture balance task

**Fig. 6:**
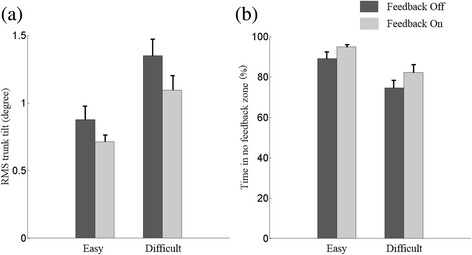
Averaged (**a**) RMS trunk tilt and (**b**) percentage of time inside the no feedback zone across all participants with difficult exercise compared with easy exercise for Feedback Off and Feedback On conditions

The selected exercises for the participants are shown in Table 1. Mean RMS trunk tilt was smaller with feedback than without feedback (F(1,3) = 72.007, *p* = 0.003) and the percentage of time inside the no feedback zone was larger with feedback than without feedback (F(1,3) = 31.395, *p* = 0.011) (Fig. 6). The RMS tilt was larger (F(1,3) = 16.651, *p* = 0.027) and the percentage of time inside the no feedback zone was smaller (F(1,3) = 17.054, *p* = 0.026) with difficult exercise than easy exercise. Significant interactions between feedback and difficulty of exercise was not found for RMS tilt (*p* = 0.094) or the percentage of time inside the no feedback zone (*p* = 0.255).

**Table 1:** The selected exercises for the participants

Participant ID	Easy exercise	Difficult exercise
P1	Eyes closed, semi-tandem Romberg, foam surface	Eyes closed, Romberg, foam surface
P2	Eyes closed, semi-tandem Romberg, arm crossed, firm surface	Eyes closed, semi-tandem Romberg, foam surface
P3	Eyes closed, semi-tandem Romberg, arm crossed, firm surface	Eyes closed, semi-tandem Romberg, foam surface
P4	Eyes closed, semi-tandem Romberg, arm crossed, yaw head movements, firm surface	Eyes closed, semi-tandem Romberg, foam surface

### Gait retraining task

**Fig. 7:**
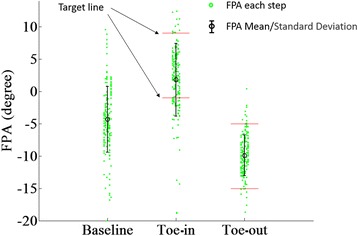
Gait retraining task results. Green dots represent the FPA for each step and each column of data corresponds to FPA values for a given participant. No feedback was given between the red target lines. Toe-in FPA was significantly larger than baseline and toe-out (*p* < 0.01), and toe-out FPA was significantly smaller than baseline (*p* < 0.01)

The real-time wearable feedback system enabled participants to significantly change their FPA for toe-in and toe-out gait patterns (Fig. 7). There were significant differences between baseline walking, toe-in gait and toe-out gait (F(2537) = 273.8, *p* < 0.001). FPA during toe-in gait was significantly larger than baseline walking (p < 0.001) and toe-out gait (p < 0.001), and FPA during toe-out gait was significantly smaller than during baseline walking (p < 0.001). During toe-in gait retraining, participants walked inside the no feedback zone 68% of the time, received feedback indicating more toe-in needed 25% of the time, and received feedback indicating less toe-in needed 7% of the time. During toe-out gait retraining, participants walked inside the no feedback zone 89% of the time, received feedback indicating more toe-out needed 5% of the time, and received feedback indicating less toe-out needed 6% of the time.

## Discussion

This study presented design and usability/feasibility testing for a wireless, configurable, wearable sensing and feedback system for real-time postural balance and gait training. Preliminary results showed that participants successfully used the system to alter their trunk movements during a postural balance task and foot movements during a gait retraining task.

In the postural balance task, the system captured the trunk tilt and provided meaningful vibrotactile feedback in the M/L direction, and healthy older adults could use the feedback to reduce their body sway for the balance exercises. The RMS of trunk tilt decreased by 20% and the percentage of time inside the no feedback zone increased by 10%, which is consistent with the trend of the results of prior studies using other systems [27, 34, 36]. Haggerty et al. [36] found that older adults could use a high fidelity laboratory-based system (Xsens MTx and C-2 tactor) to increase the percentage of time inside the no feedback zone from 65% to 90%. While the presented system increased the percentage of time inside the no feedback zone in a way that was similar and consistent with previous work, the extend of this increase was not as pronounced as other high fidelity systems [28], likely because of the lower fidelity sensors, lower sampling and algorithm update rates, and longer spin-up time of the flat eccentric vibrotactile feedback motor (resulting in longer delays as compared to linear actuators [29]) in the presented system as compared with high fidelity systems.

In the gait retraining task, toe-in and toe-out FPA were significantly different compared with baseline values. The mean toe-out FPA roughly corresponded with the middle of the no feedback zone (Fig. 7) and the percentage of time inside the no feedback zone was relatively high (89%), suggesting that the no feedback zone was easily found and maintained by participants during toe-out gait. Mean toe-in FPA in contrast was not as near the center of the no feedback zone (Fig. 7) and the corresponding percentage of time inside the no feedback zone was relatively lower (68%). Also, during toe-out gait, the amount of feedback indicating more toe-out was approximately equal to the amount of feedback indicating less toe-out, but during toe-in gait, feedback was more than 3 times as likely to indicate more toe-in needed than less toe-in needed. This suggests that it was relatively more difficult for participants to maintain a large toe-in than a large toe-out gait, which may be due to physical limitations, discomfort, or the inability to perform large toe-in gaits [4].

Compared with traditional training systems, limited by tethered wires and stationary cameras or wearable systems which are generally only designed for a single application, the presented system is portable and configurable for both postural balance and gait training. Simic et al. [36] used a laboratory system with real-time visual feedback to effectively train a variety of different FPAs for knee osteoarthritis patients. Other similar approaches with laboratory systems using real-time visual or haptic feedback have also been shown to train various FPAs for healthy participants and knee osteoarthritis patients [3, 37, 38]. The presented wearable system provides similar functions as laboratory-based training systems and more flexibility for rehabilitation and training outside of the laboratory. In this paper, trunk sway and foot progression angle calibration are simple and each last less than 10 s and calibration was provided at beginning of each trial, however other human movement parameters may require longer calibration times [39], and in the case of large numbers of sessions/subjects, sometimes any calibration (even if short) can be detrimental. Another option could be on-the-fly calibration methods that automatically calibrate during the initial phase of testing [40, 41]. The total delay of the control loop was 120 ms, which is less than the typical human reaction time to vibrotactile stimuli [42, 43] and was thus considered acceptable for real-time training. The configuration of the vibrotactile feedback nodes is limited by the spatial and temporal resolution of skin. For example, The distance between the feedback nodes should larger than the known spatial resolution based on vibrotactile perception for any given body segment (e.g. 40 mm on the limbs [44] and 20-30 mm on the trunk [45]), and minimum temporal resolution requirements need to be considered [46].

The battery life of each Dot is roughly 1.5 h, which is long enough to meet most rehabilitation demands but could be insufficient for longer training sessions. Most energy is consumed by the motors and ZigBee module, which in the future could be replaced by linear actuators [47] and Bluetooth Low Energy networking (BLE) [48] for lower energy consumption. Because BLE modules are typically designed for one-to-one communication, most multi-channel body area networks rely on the ZigBee protocol [49]. However, newer BLE modules may satisfy simultaneous multi-channel communication requirements and should thus be further explored. Higher capacity and more efficient batteries could also extend battery life. While the vibration frequency was set near the peak human skin sensitivity in both experiments, the vibration frequency can also be changed as needed. The usability and feasibility experiments performed were preliminary in nature and focused on demonstrating this system as a proof-of-concept. Two participants were excluded from this study due to the inability to internally rotate their foot far enough. Therefore, the presented system is not well suited for persons with extreme muscle weakness or sensory impairments like spinal cord injuries, where assisted guidance of a therapist or robot is required [50]. As this study is only meant to be a proof-of-concept, we chose to perform two separate pilot studies with two configurations of sensor and feedback devices for two separate movement activities (standing posture and walking gait). However, future research is needed to further explore a wider range of movement activities for human assessment and training. Both experiments were performed with healthy older adults for a single session and therefore, the effects of motor learning and rehabilitation have not yet been fully assessed. Future research is needed to explore the possibility of learning and retention through multiple training sessions.

Because rehabilitation effectiveness is often limited by patients’ inability to frequently travel to a clinic to perform repetitive rehabilitation tasks and the lack of controlled home exercise programs outside of clinic settings [51], long-term, home-based rehabilitation is a potential alternative and has been shown to mitigate the risk of reduced mobility during daily living activities and to improve the recovery rate [52, 53]. Thus, the presented wearable system could potentially be used for long-term, home-based rehabilitation applications as an alternative to laboratory-based systems. As this study was preliminary in nature, the number of participants was small and the strength of the experimental results somewhat limited, thus future research should focus on more participants to strengthen the findings.

## Conclusion

This paper describes a wearable sensing and feedback system for postural and gait training. We performed usability and feasibility testing and demonstrated that the wearable system can be used to measure, provide real-time feedback for, and effectively train human movements during postural balance and gait tasks. It is possible that the system could be used for other movement training applications by implementing new sensing and feedback algorithms within the configurable software architecture (Fig. 2). In general, the system is portable, configurable and effective for real-time human movement training and thus may be well-suited for long term, home-based rehabilitation, research and treatment.

## Abbreviations

BLE: Bluetooth low energy networking
BMI: Body mass index
FPA: Foot progression angle
IMMU: Inertial measurement magnetometer unit
M/L: Medial-lateral
RMS: Root-mean-square
